# Genome Editing in Cereals: Approaches, Applications and Challenges

**DOI:** 10.3390/ijms21114040

**Published:** 2020-06-05

**Authors:** Waquar A. Ansari, Sonali U. Chandanshive, Vacha Bhatt, Altafhusain B. Nadaf, Sanskriti Vats, Jawahar L. Katara, Humira Sonah, Rupesh Deshmukh

**Affiliations:** 1 Department of Botany, Savitribai Phule Pune University, Pune 411007, India; waquar.ansari@gmail.com (W.A.A.); sonalc2022@gmail.com (S.U.C.); vacha.biotech@gmail.com (V.B.); 2 Agri-Biotechnology Division, National Agri-Food Biotechnology Institute (NABI), Mohali 140306, India; sansvats@gmail.com (S.V.); biohuma@gmail.com (H.S.); 3 ICAR-National Rice Research Institute, Cuttack 753006, India; jawaharbt@gmail.com

**Keywords:** Biotic/abiotic stress, cereals, CRISPR/Cas9, genome editing, non-GMO

## Abstract

Over the past decades, numerous efforts were made towards the improvement of cereal crops mostly employing traditional or molecular breeding approaches. The current scenario made it possible to efficiently explore molecular understanding by targeting different genes to achieve desirable plants. To provide guaranteed food security for the rising world population particularly under vulnerable climatic condition, development of high yielding stress tolerant crops is needed. In this regard, technologies upgradation in the field of genome editing looks promising. Clustered regularly interspaced short palindromic repeats (CRISPR)/Cas9 is a rapidly growing genome editing technique being effectively applied in different organisms, that includes both model and crop plants. In recent times CRISPR/Cas9 is being considered as a technology which revolutionized fundamental as well as applied research in plant breeding. Genome editing using CRISPR/Cas9 system has been successfully demonstrated in many cereal crops including rice, wheat, maize, and barley. Availability of whole genome sequence information for number of crops along with the advancement in genome-editing techniques provides several possibilities to achieve desirable traits. In this review, the options available for crop improvement by implementing CRISPR/Cas9 based genome-editing techniques with special emphasis on cereal crops have been summarized. Recent advances providing opportunities to simultaneously edit many target genes were also discussed. The review also addressed recent advancements enabling precise base editing and gene expression modifications. In addition, the article also highlighted limitations such as transformation efficiency, specific promoters and most importantly the ethical and regulatory issues related to commercial release of novel crop varieties developed through genome editing.

## 1. Introduction

Recent advancements in the genome editing technologies have supplanted the impediments of conventional breeding strategies and commenced a new crop improvement era. Site-specific nucleases (SSNs) were used for editing of genomes which changes target location of genes present in the genome. In the targeted DNA double stranded break (DSB) was created by zinc finger nucleases (ZFNs), transcriptional activator-effector nucleases (TALENs) and CRISPR-related endonuclease Cas9 (CRISPR/Cas9). Subsequent repair of DSB is carried either through homologous recombination (HR) or non-homologous end joining (NHEJ), which are cell’s specific inherent repair mechanism [1]. NHEJ mediated repair is an error prone pathway, that makes arbitrary changes either insertions or deletions (InDels) causing mutations through frame shift which usually results in the knockouts of genes. The pathway mediated through HR is considerably more precise in the process of homologous sequence exchange making it useful tool for the gene knock in or gene replacement. In recent times the most effective and easiest genome editing tool considered is CRISPR/Cas9 [2], comprising a guide RNA (small RNA fragment) that is associated with a DNA endonuclease known as Cas9. The gRNA framework comprised of two parts which include CRISPR-derived RNA (crRNA) and trans-activating RNA (tracrRNA). In nature, particularly double stranded DNA cutting site is targeted through the crRNA, which has a small homology region, enabling crRNA to bind the tracrRNA. The stem loop structure is mediated through tracrRNA that integrates along with Cas9 protein. In genome editing technique which is mediated through CRISPR/Cas9, the crRNA and tracrRNA are built into a single guide RNA chimera (sgRNA) which likewise coordinates the sequence dependent Cas9 dsDNA break. Cas9-sgRNA complex binds to the target site where sgRNA matches with the homologous sequence and creates a DSB (double stranded break) [3].

The conventional techniques, for example, hybridization, selection and hybrid breeding were implemented successfully by exploring the knowledge of genes and quantitative trait loci (QTLs) regulating different traits. The molecular breeding approaches were mostly based on the identification of molecular markers linked to the genes/QTLs, and subsequent marker-assisted selection (MAS) [4]. However, such breeding applications are completely dependent on the natural variation existing in the primary gene pool. Genome editing efforts were propelled two decades back in plants with frequencies of low targeted integration, and as a result of the revelation of novel nucleases inciting DSBs at specific loci, frequencies of genome editing was significantly improved. In maize, *AHAS108* and *AHAS109* genes (*acetohydroxyacid synthase*) were edited utilizing chimeric form of RNA/DNA oligonucleotides, and the recurrence frequency was 10^−4^ [5]. This was more than the spontaneous mutations and homologous recombination mediated genome editing [5]. Moreover, genes complicated to manipulate by regular mutagenesis was effectively covered with genome editing technique and were explored for their accepted role, for example rice ROS1, linked to cytosine DNA demethylation and consequently plants epigenetic alterations. With the progression in ZFNs, TALENs and CRISPR/Cas, all these techniques have been implemented to modify specific gene/locus in many cereal crops as presented in Table 1.

The CRISPR/Cas9 technique was effectively utilized in crops of importance more particularly cereal crops because of its acceptability, cost effectiveness, less time taken and enhanced and focused targeting [6]. The significant improvement in genome editing tools are anticipated to eradicate the flaws and concerns with the transgenic technology and expected to replace the transgenic development approach at least for the commercial release. Considering the fast growth and potential implications, number of review articles addressing genome editing and its importance in various plants have been published recently [7].

Cereals are staple food in our diet since the establishment of practice of agriculture considering the major health benefits, nutritional value and production. Complex carbohydrates are major content of cereals that provide us ample energy. Cereals provides sufficient proteins, lipids, fats, vitamins, minerals, and abundant fibers. Cereals are also the major source of iron, niacin, riboflavin, and thiamine. Also considered as primary source of energy for the human being worldwide as they provide over 20% calories of daily diet [8]. Therefore, cereals have great importance for the global food security. Considering the importance, genome editing techniques have been widely employed for the improvement of cereal crops, acquiring new possibility to advance novel varieties with enhanced produce and superiority. The present review addresses especially the exploration of recent advancements in genome editing techniques providing opportunity to develop novel cereal crop varieties with enhanced yield, stress tolerance and better nutritional quality.

## 2. Basic Approaches Used for Genome-Editing

### 2.1. Zinc Finger Nucleases

The ZFNs (Zinc finger nucleases) are designed restriction enzymes providing a powerful tool used for the genome editing. The DSBs at target are introduced by these endonucleases, followed by error-prone non-homologous end joining (NHEJ) repair which makes small deletions or insertions at the ZFN cleavage locus. ZFNs are chimeric proteins which consist of two domains one is synthetic zinc finger-based domain that binds to DNA and other is non-specific DNA cleavage domain [40]. Fok1 is typically used in the cleavage domain which belongs to the type IIS class of restriction endonucleases. It has N-terminal DNA binding domain and C-terminal domain which has nonspecific DNA cleavage activity. DNA binding domain is comprised of zinc finger domains in 3–6 number, each domain is able to recognize a target DNA sequence of 3 base pair (bp) length. A pair of Zinc finger arrays (ZFAs) binds to respective sequences targeted and get aligned in reverse fashion with each other. Binding sites of two ZFAs (each 18–24 bp in length) are separated by 5–8 bp. This spacing is a critical part of ZFN design as it allows Fok1 monomer to dimerise and create a DSB in the target sequence. The specificity of ZFNs made their application wide in targeted gene editing in plants and animals.

The genome editing using ZFNs were first reported in *Arabidopsis* and Tobacco. Subsequently, ZFNs were successfully utilized in maize, wheat, rice, and other plants. Shukla et al. [41] reported targeted cleavage of inositol-1,3,4,5,6-pentakisphosphate kinase 1 (*IPK1*) one of the phytic acid biosynthesis genes, in developing maize seeds it gives the characteristics of both herbicide tolerance and desired alteration of the inositol phosphate profile. Li et al. [42] mutated *OsCKX2* (Cytokinin oxidase 2) that increased grain number and subsequently the yield. Jung et al. [43] generated transgenic rice with modification in *SSIVa* gene which is involved in the starch biosynthesis pathway and concluded that the engineered ZFNs can affectively cleave and stimulate mutations at *SSIVa* gene in rice. Recently, ZFNs mediated gene editing was efficiently used in allohexaploid wheat in which coding sequences of acetohydroxyacid synthase (*AHAS*) was targeted to protect against imidazolinone herbicides and, this research resulting in 2.9% recovery in transgenic plants [44].

The advances in ZFN based genome editing provide enormous opportunity to target any DNA sequence in the genome. However, ZFNs have some limitations e.g., their construction is challenging, lower affinity for AT rich region and can bind to any DNA sequence other than the target site that leads to off target effect.

### 2.2. Transcription Activator-Like Effectors Nucleases

The TALEN approach has been used for precise genome editing. TALEN consists of Transcription Activator-like Effector (TALE) array fused with non-specific cleavage domain of FokI endonucleases. These effectors are Type III effector proteins discovered in plant pathogenic bacteria *Xanthomonas* that infects plants rice and cotton [45]. The TALEN assembly consists of a central DNA binding domain, nuclear localization signal (NLS), highly conserved acidic transcription activation domain (AD) at C terminal and secretion and translocation signal at N terminal. Central DNA binding domain consists of 33–35 amino acid tandem repeats; each repeat recognizes one nucleotide in the target sequence [46]. Thus, designing of TALEN is respectively easier than ZFNs. The specificity of TALEN is determined by amino acids of polymorphic nature called as repeat variable di-residue (RVD) which are located only at 12 and 13 positions. Many RVDs have been described in the literature; major RVDs being NI (Asn Ile), HD (His Asp), NN (Asn Asn), NK (Asn Lys) and NG (Asn Gly) which recognize nucleotides Adenine (A), Cytosine (C), guanine (G), thymine (T) respectively. These tandem repeats are followed by sequence of 20 amino acids, which are known as half repeats. Mechanism of action of TALEN is similar to ZFNs. When two TALENs are delivered in the cell through type III secretion mechanism, they are translocated to the nucleus and bind to the target DNA strand in an opposite orientation with spacing of 12–30 bp and then mimics host transcription factors to reprogram gene expression of the host [47]. After binding of TALENs, FokI dimerizes and cleaves at spacer region leading to DSB in the target.

The TALEN mediated genome editing technology has been used in several plants *Arabidopsis*, rice, barley, soybean and maize to improve qualitative and quantitative traits as well as to identify the role of several genes whose functions remain unknown. For example, when knocked-out the *NDUFA9* (*NADH dehydrogenase1α subcomplex 9*), nuclear gene was identified as an assembly factor that stabilizes the junction between the membrane and the matrix arm in the mitochondrial respiratory chain complex I [48]. This editing technology in plants was first reported in rice where promoter region of *OsSWEET14* gene was disrupted which resulted in resistance to *Xanthomonas oryzae*. Zhang et al. [49] reported TALEN system efficiency in rice by evaluating transferability of TALEN-mediated mutations and observed critical features of TALEN techniques. The TALEN backbone N287C230 come with less mutation frequency (0–6.6%), however C-terminal domain truncations considerably enhances 25% efficiency. In most transgenic T0 plants, TALEN delivered a solitary common mutation joined by an assortment of low-recurrence mutations. For every individual T0 plant, the pervasive mutation was available in many tissues inside a solitary tiller just as in all tillers inspected, recommending that TALEN-actuated mutations happened very early in the emergence of the shoot apical meristem. Multigenerational investigation demonstrated that TALEN induced mutation was steadily transmitted to the T1 and T2 populations in a typical Mendelian manner. Haun et al. [50] reported improved soybean oil quality by mutagenesis in fatty acid desaturase 2 gene family. TALENs are preferred over ZFNs because of easy and quick assembly, high success rate, availability of powerful resources and less off target effects. Though the TALEN is a powerful technique, it has some limitations it cannot edit methylated target site, structural limitation being that the recognition code should always be preceded by a thymine nucleotide and its big size because of which its delivery with a vector is problematic and challenging. To overcome this, three platforms are used for assembly: 1. Standard cloning assembly 2. Golden gate assembly and 3. Solid phase assembly method.

### 2.3. CRISPR/Cas9 Based Genome Editing

The CRISPR approach is the most recently invented and most efficient technology for genome editing. Ishino et al. [51] discovered the CRISPR such as repeats and the initial characterization of CRISPR-Cas system was performed in the 1990s. Later, it was Jansen et al. [52] who coined the term CRISPR. It is an adaptive immune system of bacteria and archaea which protect them from invading viruses. Cas systems are divided into 2 classes, 6 types and 19 subtypes according to the structure [53]. The major variation between the classes is the composition of the effector nuclease. The effector of Class I is composed of several proteins having different functions which form a complex, whereas the effector of Class II is composed of a single protein with multi-domains [54]. Type II-A CRISPR/Cas9 is the largely used system in which Cas9 effector, spCas9 is isolated from *Streptococcus pyogenesis* due to its higher efficiency of generating DSB. The spCas9 showed some restrictions such as the protospacer adjacent motif (PAM) is NGG (N-Any nucleotide, G-Guanine) resulting its application difficult in AT rich sequences and it is also prone to produce off target effects. To overcome these limitations, variants of cas9 have been obtained in which high fidelity Cas9 holds mutations that decreases the interaction between non-specific DNA and nuclease domain which ultimately reduces off target effects [55]. CRISPR/Cas system mainly consists of three stages; expression, interference and adaptation. In the course of expression of CRISPR array, which carries sequences which shows homology to target sequences (protospacers), which gets transcribed in pre-CRISPR RNA (pre-crRNA) then these pre-crRNA form homologous bonds with trans activating crRNAs (tracrRNA). When pre-crRNA/tracrRNA complex is formed, it attaches to the Cas9 protein and here the long pre-crRNAs are cut by RNase III into individual crRNA/tracrRNA complexes (gRNA). Interference begins when crRNA/tracrRNA guides the Cas9 complex to target sequence and gRNA binds to the target sequence after PAM. The PAM sequence is absent in CRISPR array and therefore, this sequence allows self/non-self discrimination. Cas9 has helicase and nuclease activity therefore target sequence is unwound and cut by RuvC and HNH domain of Cas9 protein leaving DSBs in the target. This DSB is repaired by NHEJ or HDR. The repaired sequence is transcribed and adapted into the genome [56]. CRISPR has many advantages over ZFNs and other technologies such as it is simpler, cost effective and easy to construct as Cas9 is readily available that makes it as an attractive genome-editing tool. Multiple genes in the genome could be targeted simultaneously using multi target approaches. Cas9 can be converted into nickase by mutating RuvC domain which ultimately reduces off target effects. The CRISPR system has been used in wide range of crops to improve quality and nutritional value of food, higher yield, biotic and abiotic stress tolerance (Table 2). Cataloging of published studies suggest that the CRISPR techniques can be implemented to target any gene of interest [17]. A seminal study by Shan et al. [13] demonstrated the genome modification of three rice genes, namely, mitogen-activated protein kinase (*OsMPK2*), phytoene desaturase (*OsPDS*) and betaine aldehyde dehydrogenase2 (*OsBADH2*) through CRISPR/Cas9 for the first time. Later many reports in other crops such as barley, maize, wheat, and soybean were published (Table 2).

Kim et al. [57] described about CRISPR/Cas9 based wheat protoplasts genome altering system for abiotic stress linked two genes, in particular, wheat dehydration responsive element binding protein 2 (*TaDREB2*) and wheat ethylene responsive factor 3 (*TaERF3*). Almost 70% of protoplasts were transfected effectively and expression of these altered genes were affirmed with the T7 endonuclease study. Present DNA-free editing technique maintains a strategic distance from tedious methods, for example, backcross breeding for the expulsion of the transgene and permits to acquire transgene-free plants at T0. Feng et al. [11] shown the application of the CRISPR/Cas9 techniques in maize by focusing on the gene linked with albino marker, *Zmzb7* in a protoplast system. *Zmzb7* knockout brings albino plant, with the sgRNA intended to focus in the eighth exon region of *Zmzb7*, while maize U3 promoter were utilized for expression. Subsequently, maize embryos transformation mediated through agrobacterium, 31% mutation efficiency was shown by T0 lines. The CRISPR/Cas9 system efficiency to induce selective mutation and the heritability in mutant lines have been studied by targeting genes such as *OsPMS3*, *OsMSH1*, *OsDERF1*, *OsEPSPS*, and *OsMYB5* [58]. Discrepancy in rate of mutation (21–66%) was recorded in the T0 generation for number of genes with no or 1 bp off-target mutation. Directed base changes for the herbicidal gene, *C287* in rice was made conceivable utilizing activation induced cytidine deaminase (Target-AID) technique [59] in which dCas9 attached with cytidine deaminase was utilized for base altering without generating DSBs. Correspondingly, Zong et al. [60] exhibited an exact genome altering in rice, wheat and maize. Li et al. [61] exhibited base altering of rice *OsPDS* and *OsSBEIIb* genes utilizing BE3 base editor.

## 3. Precise Genome Editing in Cereals

The gene transfer strategies applied in the crops that are refractory to effective transformation is named as in planta gene transfer. In this technique, template for repair and CRISPR/Cas9 construct are transformed stably into the genome of the plant. The transformed CRISPR/Cas9 construct expressing Cas9 nuclease makes cuts at the target and simultaneously, DNA repair utilizing the transformed template takes place with the inbuilt homology directed repair (HDR) mechanism. In this process Cas9 plays dual role, first to make DSB at target site as well as excise out the template to activate the HDR mechanism. Endo et al. [34] reported CRISPR/Cas9 mediated sequence specific genomic editing of 3 rice genes which includes mitogen-activated protein kinase (*OsMPK2*), phytoene desaturase (*OsPDS*) and betaine aldehyde dehydrogenase (*OsBADH2*) genes, those are concerned with regulating reaction against number of abiotic stress stimuli. In transformed cells, the constraint of decreased measures of HDR template can be tended by utilizing geminivirus replication mechanism to expand copy number of repairs. Utilizing this methodology, it was conceivable to accomplish the effective gene transfer by means of transformation of cotyledons or leaf explants mediated through *Agrobacterium* and consequent regeneration in tomato plant [74,75].

Biolistic transformation is another strategy useful for the delivery of larger repair template which is usually difficult with the transformation mediated through *Agrobacterium*. The site-specific gene alterations and insertions were shown in soybean [76], and maize [77] by employing recovery of immature embryos and biolistic transformation. Gil-Humanes et al. [78] joined viral replication with biolistic transformations of wheat cells and accomplished multiplexed gene transfer at all three homeo alleles. Rather than HDR, the NHEJ additionally can be utilized for the exact genome altering. For instance, gene replacements and additions were shown in the rice 5-enolpyruvylshikimate-3-phosphate synthase gene (EPSPS) by focusing on two neighbouring introns and furnishing a repair template having point mutations in the exon of intermediate characteristics by means of biolistic transformation [23].

## 4. Genome Editing Specificity

**Figure 1:**
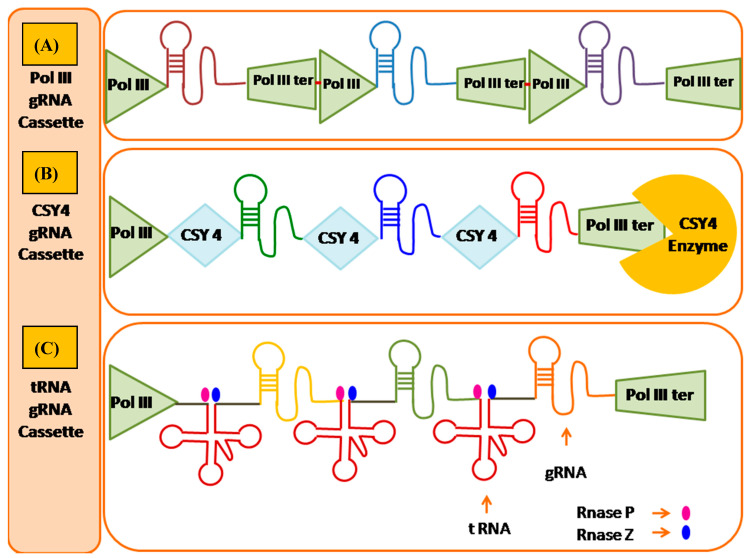
Overview of various approaches for multigene targeting using CRISPR/Cas9 technology. (**A**) Pol III-gRNA cassette comprises Pol III promoter followed by SgRNA and Pol III terminator respectively. Here each SgRNA uses separate promoter and terminator for expression. (**B**) CSY4-gRNA cassette contains repeated units of CSY4 and different conserved gRNAs. At the end CSY4 endonuclease gene (yellow in color) also cloned in the vector which cleaves at restriction sites present in CSY4 (blue color rhombus) that ultimately separates various SgRNAs, which can further proceed for genome editing. (**C**) tRNA-gRNA Cassette comprises alternate units of eukaryotic Pre-tRNA (red color clover leaf structure) and conserved gRNAs (different colored structures), that is cleaved by RNaseP (pink circle) and RNaseZ (blue circle) to liberate mature tRNAs. These separated gRNAs can proceed for double strand break by interacting with Cas9 cloned in the same vector.

The genome editing through CRISPR/Cas9 approach is rather very precise for manipulating target sites but still has concerns about the off-target mutations. The off-target mutations are more frequent with the plants having higher ploidy levels and also for the genes with many paralogs. In plants, higher specificity was confirmed by Feng et al. [11], as CRISPR/Cas9 induced mutants whole genome was sequenced, and perhaps no off-target effects were distinguished. Though, reports are available showing off-target cleavage in soybean, maize and rice, mostly happening in gene paralogues with the sequences which are nearly the same to the targets [24]. By applying computational sgRNA selection, protein engineering, modifications of RNA and improved systems of construct delivery, editing specificity can be enhanced [79,80]. The use of bioinformatics tools, optimization and advancement for designing very specific gRNA of interest, and identifying the possible off-target editing sites permits considerable increments in specificity of the CRISPR/Cas system. The genome-wide designing of very specific sgRNAs with limited off-target impacts, performed in eight plant species showed that designing of minimum 10 such sgRNA is possible in 67.9–96.0% genes, except for maize where just 30% of transcripts permits at least 10 designs sgRNA [31]. However, simultaneous editing of paralogs having high level of sequence similarity can be possible with a common sgRNA. This is particularly important in polyploid crops such as wheat and canola where multiple homeo genes alleles can be editing with same sgRNA. Additional solution dealing with the concern of off-target action include the application of truncated sgRNAs and paired nickases [48]. The off-target editing was also affected due to the dosage of Cas9 and sgRNA, off-target cleavage increases with elevated enzyme concentrations. For example, to express sgRNAs Ranganathan et al. [81] utilized the more fragile H1 promoter, bringing down the off-target impacts. Off-target cleavage is such aswise influenced by various delivery techniques, in rice and wheat genome editing was achieved effectively, recently by delivering a pre-assembled RNP complex of Cas9 and gRNA into embryos of immature nature [17]. The idea of CRISPR/Cas system makes it profoundly manageable through multiplexing approaches, by simultaneously focusing on the genomes multiple sites. Even though it is achievable to use multiple different promoters [82], the effectiveness of this methodology decreases with expanding size of construct, due to this it is applicable for guides in small number. The endogenous tRNA processing machinery were utilized by Xie et al. [80] for the expression of sgRNAs in multiple number from a single synthetic gene comprising multiple repeats of sgRNA and tRNA. Cleavage by the tRNA-processing RNases of endogenous nature, results in the release of individual sgRNAs, at the same time eight genes has been edited so far by utilizing this methodology (Figure 1).

## 5. Predicted Boom over Coming Years

CRISPR/Cas drastically builds the possibility to get better characteristics in different crops when match up with breeding practices being conventionally carried. Crop improvement of selected germplasm can be achieved in single generation by homozygous pyramiding of genes of interest. Indistinct from natural allelic variations CRISPR/Cas is such aswise an amazing tool that introduces heritable changes for particular traits. In addition, when the CRISPR/Cas construct transformed in to plant, it will get inserted in the genome randomly, mostly on different locus or chromosome other than the target site. And the transgenic plants obtained are hemizygous for the insert as well as mutated target genes. This implies that crossing or selfing these plants can produce transgene free progeny, diminishing the possible dangers and administrative necessities which may be applied for the transgenic crops. Knockout of negative regulator genes through CRISPR/Cas9 is much easier to achieve desirable manipulations in the trait (Table 1). For example, CRISPR/Cas9 was utilized to improve blast and bacterial blight resistance in rice by altering the negative regulators such as translation factor related to ERF and the *SWEET* (sugars will eventually be exported transporter) genes, respectively [26]. For the improvement of crops, the biotechnology industry effectively implemented and utilized genome editing techniques. To get an amino acid exchange, transgene free, rice blast disease tolerant rice was developed using *OsERF922* gene by utilizing an exclusive genome editing system [26].

## 6. CRISPR/Cas9 Based Genome Editing in Cereals

### 6.1. Priority Traits

In cereal crops large number of genome editing was reported targeting various biotic and abiotic stress, nutritional and yield related traits (Table 1). The principle target of genetic improvement of cereal crops is to enhance food productivity and provide nutritional security to the growing population. Various traits which have been targeted through CRISPR/Cas9 based genome editing in cereal crops mainly include improvement of resistance against diseases caused by bacterial, fungal, insect and viral pathogens. Additionally, concern towards genome editing targeting herbicide tolerance and nutritional quality improvement were also in the top priority.

#### 6.1.1. Resistance Against Bacterial Disease

Many plant diseases are caused by bacteria and they also produce numerous metabolites which include toxins, polysaccharides, pectic compounds, and also hormones. Very few reports are published which presents implementation of CRISPR/Cas system to enhance resistance against bacterial disease. In rice mutagenesis of *OsSWEET13* through CRISPR/Cas9 was performed to enhance resistance against bacterial blight caused by *Xanthomonas oryzae* pv. *oryzae* [83]. A susceptibility gene *OsSWEET13* encodes a transporter of sucrose which participates in plant-pathogen interaction. PthXo2X, an effector protein produces by oryzae increases expression of *OsSWEET13* in the host. In earlier report, TALEN approach based mutagenesis resulted in disruption of *OsSWEET14* gene make *X. oryzae* effector not capable to bind with *OsSWEET14*, resulted in resistance against disease [42]. Advance genome editing strategy for multiplexed recessive resistance through an amalgamation of the foremost effectors and further resistance (R) genes will be a future aspect to achieve bacterial blight resistance. Crown gall tumors are generated when pectin layers in plant cells are degraded by pectic enzymes. In this regard, *CsLOB1* genes of *Citrus* targeted by CRISPR/Cas9 approach [84] showed enhanced resistance against the *Xanthomonas axonopodis* bacterium. Additionally, in homozygous mutants developed from *Citrus* explants, deletion of the whole effectors-binding components (*EBEPthA4*) succession from both *CsLOB1* alleles presented a high level of bacterial obstruction. In rice, mutagenesis of *OsSWEET13* was performed by Zhou et al. [83] by utilising CRISPR/Cas9 approach to develop bacterial blight resistance.

#### 6.1.2. Resistance against Fungal Disease

Present day agriculture is reliant on chemical compounds to prevent loss due to diseases caused by fungus. The appliance of chemicals thorough farming impacts human health and the environment. Focusing development of wheat cultivars resistant to fungal diseases has been a noteworthy goal in recent times. Specifically editing of genome to knock-out the genes that regulate fungal pathogen resistance in wheat [14]. With the help of CRISPR/Cas9 and TALEN technique, *TaMLO-A1*, *TaMLO-B1*, and *TaMLO-D1* genes were concurrently targeted to achieve resistance agaist powdery mildew. In the same way, resistance to Fusarium head blight (FHB) were also targeted in wheat by selecting three different wheat genes including the nuclear transcription factor *X box-binding such as 1* (*TaNFXL1*), an *ABC transporter* (*TaABCC6*), and a gene which encodes a nonspecific lipid transfer protein (nsLTP), *TansLTP9.4* [85]. CRISPR/Cas9 based genome editing was practically implemented in developing japonica rice resistance against blast disease through targeting translation initiation codons of *OsERF922* to introduce InDels [86]. These mutant lines were further evaluated for different agronomical traits such as leaf width and length, percentage of seed setting, panicle length, number of fertile tiller, plant height, and thousand seed weight, and none of the reported traits showed significant different from wild-type plants, explaining that changes in *OsERF922* may result in plants with enhanced resistance without any negative impact on plant development. Blast disease resistant rice plants were developed using CRISPR/Cas9 employing disruption of rice genes *OsERF922* and *OsSEC3A* [87]. *OsSEC3A* mutated plants interrupted in complex putative subunit which participates in exocytosis, open a pleiotropic phenotype includes enhanced disease resistance against *Magnaporthe oryzae*, increased salicylic acid (SA) levels, and induction of pathogenesis- and SA linked genes [87]. On the contrary, no changes in numerous agronomic parameters were recorded in T1 and T2 transgene free plants having mutated ethylene responsive factor (ERF)922 gene, which is a transcription factor concerned in numerous stress reactions.

In case of bacterial as well as fungal diseases, pathogens evolved rapidly and overcome the vertical resistance in the host. Therefore, resistance developed through targeting single or few genes by CRISPR/Cas9 approach may not last for many generation. In this case multiplex genome editing paved the way to develop a mechanism which can overcome the resistance developed in fungus and bacteria, employing multi-gene targeting strategy [88]. In multiplex genome editing, multiple guide RNAs which target various genomic sites is simultaneously used [20]. For example, number of genes linked with phenotypic alterations was selected such as Stromal Processing Peptidase (*SPP*), Rice Outermost Cell-specific gene5 (*ROC5*), and Young Seedling Albino (*YSA*) in rice and positive results was obtained [20]. Similarly, studies performed in maize [62], and wheat [14] has developed a foundation for the application of CRISPR for multiple gene editing in cereals and other crop plants. Fascinatingly, three distinct groups [13,25,89] showed the establishment of rice and tomato T1 generation biallelic or homozygous mutations, presents the increased efficiency of multiplex genome editing. These alterations at genetic level are segregated usually in succeeding generations with no further modifications [89]. CRISPR/Cas9 system is constantly being improved for increased effectiveness and specificity of gene targeting. Above discussed strategies could be employed to develop tolerance against fungal and bacterial disease targeting multiple gene to overcome the issue related with breakdown of resistant against fungal and bacterial disease over the time. In continuation to this researcher assemble multiple resistance gene cassettes using single plasmid, and further introduce cluster of resistance gene at a single gene position by transformation of plant [90,91], technique is known as molecular stacking. Zhu et al. [92] stacked 3 potato late blight resistance genes *Rpisto1*, *Rpi-vnt1.1*, and *Rpi-blb3* by *Agrobacterium* mediated susceptible potato genotype transformation. Present findings represent the ease and usefulness of molecular stacking technique that could be employed for broad-spectrum disease resistance [93].

#### 6.1.3. Resistance against Insect

Insecticides being applied at large scale in agriculture field is constantly imposing threat to human health as well as ecosystem. To overcome the issue, now agriculturists are proceeding towards control of insects through biological methods and cultivation of insect resistant crop cultivars. Among very few reports where genome editing has been employed to achieve insect resistance include a study performed in cotton [94]. In this study, a mega nuclease were tried for specific cleavage of *5-enolpyruvylshikimate-3-phosphate* (*EPSPS*) and *p-hydroxy-phenyl-pyruvate dioxygenase* (*HPPD*) an endogenous tolerance genes, which results in stacking of insect-resistance and herbicide-tolerance [94]. Many research group have targeted the genome editing of disease causing insect through sex specific gene targeting. However, genome editing techniques based on CRISPR greatly revolutionized insects functional genomics. Advanced genome editing techniques are rapidly being developed by researchers. For example, site specific chromosomal translocation, DNA base editors, homology assisted genome knock in (HACK) systems and gene drives, made it possible that, we may soon be able to reduce or eradicate wild disease transmitting insects.

#### 6.1.4. Resistance against Viruses

Almost all the crop plants get infected by viruses and therefore severe losses occur in agriculture production worldwide. Chemically managing viruses is very tough due to its stringent intracellular nature of pathogenesis. Mostly, insect vectors spread the virus infection in crops and to avoid the spread, extreme pesticide appliance is very much essential. Although, the pesticide application is not sufficient to reduce the losses. Therefore, through traditional breeding many varieties with complete virus resistance were developed. Such varieties have very short span of resistance since viruses get evolved very quickly. Therefore, advance approaches need to be used to achieve quick development of virus resistant crop plants. In this regard, genome editing approaches look promising. Tomato yellow leaf curl virus (TYLCV) coding and non-coding sequences were specifically focused while intended through sgRNAs to develop virus resistance in *Nicotiana benthamiana* plants. By inducing mutations in TYLCV genome through CRISPR/Cas9, in *N. benthamiana* transgenic plants were produced which reduced or decreased viral DNA accumulation, with no or reduced indications of viral disease symptoms [95]. Recently, Macovei et al. [31] through mutagenesis of *eIF4G* alleles in rice plants, generated new resistance sources against rice tungro spherical virus (RTSV). Transgene free T2 plants obtained showed RTSV-resistance and not exhibited any demonstrable mutation in the off-target sites. However, to target in the host genome, key candidates are the translation initiation factors. Infection spreading through the viruses can be effectively checked, by altering the particular host genes which encodes responsive factor which is required by the viruses.

#### 6.1.5. Resistance and Tolerance against Herbicide

One of the pioneer study using ZFN system where herbicide (imidazolinone and sulfonylurea) resistance was achieved by targeting *SuRA* and *SuRB* (*acetolactate synthase*) genes in tobacco. Herbicide tolerant wheat variety was developed by Zhan et al. [96] through base editing performed to modulate *acetolactate synthase* (*ALS*) and *acetyl-coenzyme A carboxylase* genes that confer tolerance against herbicides such as sulfonylurea, imidazolinone and aryloxyphenoxy propionate. Mutation in *SuR* loci providing herbicide-resistance were brought effectively by using ZFN. In Arabidopsis, *5-enolpyruvylshikimate-3-phosphate* (EPSPS) gene was effectively edited, by utilizing single-stranded oligonucleotides, TALEN, and additionally CRISPR/Cas9. Efficiency of the genome editing was found to be improved with the simultaneous delivery of single-stranded oligonucleotides with TALEN or CRISPR/Cas9 segment [97]. After successful demonstration of use of single-stranded oligonucleotide to improve genome editing efficiency in model species the same approach was used in crop plants [97]. The notable example is in flax where ESPS gene was targeted using single-stranded oligonucleotides and CRISPR/Cas9. Another elegant approach is use of geminivirus replicons which was utilized in potato plants, to change CRISPR/Cas9 parts on the acetolactate synthase 1 (*ALS1*) gene targeted site and transgenic free plants mutated demonstrated decreased herbicides susceptibility. In cereal crops the most accepted and widely applied traits of agricultural important considered recently is herbicide tolerance. To achieve long term weed and insect control, it has to be priority that crops should be engineered with multiple herbicide tolerance and insect resistance genes.

#### 6.1.6. Enhanced Quality and Yield

In cereals, numerous work has been conducted to edit the genes related to quality and yield enhancement by employing CRISPR/Cas9. Shi et al. [12] conducted the precise modification of genomic DNA at the *ARGOS8* locus employing CRISPR/Cas9 in maize. The *ARGOS8* variants showed elevated *ARGOS8* transcripts. Field experiments demonstrated enhanced grain yield in *ARGOS8* variants of maize. Biofortification is considered as a sustainable approach to alleviate micronutrient deficiencies. Connorton et al. [15] reported increased iron content of wheat through genome engineering of *TaVIT2* gene. In rice, amylase content was enhanced by targeting *SBEIIb* gene [29]. Similarly, potassium deficiency tolerant rice was developed through *OsPRX2* gene-editing by Mao et al. [32]. Increased length and yield of rice plants were also obtained through gene editing of *IsPPKL1*. Heterosis is the major concern for rice and wheat. Genes known to improve heterosis such as *Gn1a* and *Gs3* can be targeted to achieve higher heterosis. Recently Huang et al. [98], reported four different yield related rice genes, *Gn1a*, *DEP1*, *GS3*, and *IDEAL PLANT ARCHITECTURE1* (*IPA1*) which were edited using CRISPR/Cas9. Similarly, numerous genes regulating yield-component traits such as number of panicles per plant, kernel weight, and number of kernels per panicle/pod/bear were targeted using CRISPR/Cas9 approach [99]. To develop high yielding genome edited plants, wheat genes were targeted with CRISPR/Cas9 such as *TaGASR7*-linked with grain length and dense-erect panicle, and plants with mutations in six alleles were generated, which showed increased thousand-kernel weight in wheat plants [64]. Increased seed size and thousand- kernel weight were shown by knockout mutants of wheat genes developed through multiplexed genome-editing [100]. The reports demonstrating use of CRISPR/Cas9 to achieve ideotype by targeting many genes simultaneously in cereal plants is expected to increase rapidly over the coming couple of years.

### 6.2. Other Traits

Genome editing has been adopted for different traits in cereal crops through insertion, deletion, and gene replacement mutagenesis. Shan et al. [13] reported higher mutation frequency through CRISPR/Cas9 system as compared to TALENs when worked with three different rice genes named *OsBADH2, Os02g23823,* and *OsMPK2*. Feng et al. [11] targeted three rice genes simultaneously using CRISPR/Cas9 construct which includes *stromal processing peptidase* (*SPP*), *rice outermost cellspecific gene 5* (*ROC5*), and *young seedling albino* (*YSA*). In barley, Lawrenson et al. [39] used RNA-guided Cas9 nuclease to edit the two copy of *HvPM19* gene simultaneously and obtained the plants with dwarf phenotype traits. Targeted gene knockout was demonstrated in maize on the endogenous *phytoene synthase* (*PSY1*) gene by Zhu et al. [5], with the relatively increased rate of mutation in transgenic plants of T0 stage, which also exhibited that the germ cells mutations can be transmitted with high efficiency to the next generation. For stable and enhanced production of rice in different drought prone areas, researchers focused through employing CRISPR/Cas9 techniques targeting leaf morphology related genes, which is a critical agronomic traits which helps in rice sustained production even under drought stress condition. *Semi-rolled leaf1,2* (*SRL1* and *SRL2*) genes mutant plants were developed by CRISPR/Cas9-based mutagenesis and the hybrids developed using mutant showed enhanced number of panicle, grain and yield per plant [101]. Such genome editing in hairy root system provides opportunity to study root development, nodule formation, and root related diseases. For target gene *inositol oxygenase* (*inox*) and *phytoene desaturase* (pds) mutations in wheat cell suspension culture RNA-guided genome editing was conducted by Upadhay et al. [99], and the mutated plants with targeted traits were cultivated in next generation.

## 7. Genome Editing for Well Characterized Genes

### Genes Previously Characterized by RNAi

RNAi (RNA interference) is a method of RNA based post-transcriptional silencing of a gene by introducing short double stranded RNA (dsRNA) complementary to target mRNA which degrades mRNA and ultimately stops protein synthesis. This technology has been used to characterize gene function and to obtain desired characteristics i.e., enhancing crop yield, quality and resistance against abiotic and biotic stresses. Earlier annotated gene targeting is critical for two important understanding, first one as still CRISPR/Cas is in initial stages of its utilization which needs validation studies, and another one is to overreach the rigorous and strict regulatory issues being elevated for the market release of RNAi technology mediated developed transgenic varieties. As by United States government genome edited crops carrying no any foreign DNA have already been declared as non transgenic crops, and the similar tag being expected from other countries additionally. Consequently, for smooth, affordable, and economically effective release of improved varieties of crop, CRISPR/Cas system is being preferred over RNAi. Qiao et al. [102] generated semi-dwarf plants from taller rice variety QX1 by RNAi suppression of *OsGA20ox2* gene which encodes regulatory enzyme GA 20-oxidase for the synthesis of biologically active gibberellic acid (GA). Gothandam et al. [103] reported knock out mutation of *OsPRP3* transcript for functional analysis of *OsPRP3* gene. This experiment suggested the cell wall protein nature of *OsPRP3* which determines floral organs extracellular matrix structure as well as in cold tolerant plants it increases cell wall integrity. RNAi mediated down regulation of *betaine aldehydedehydrogenase2* (*BADH2*) gene in non-scented rice induces expression of 2-Acetyl-1-Pyrroline (2AP) which is a principle aroma compound [104]. RNAi was used to downregulate two isoforms of starch-branching enzymes II (SBEIIa and SEBIIb) in wheat and rice endosperm to raise amylose content. The advantage of targeting genes previously annotated with RNAi or other transgenic approaches such as T-DNA insertion mutation can be used to target by CRISPR/Cas9 which will by-pass the transgenic regulatory issue associated with techniques such as RNAi.

## 8. Advances in CRISPR/Cas9 Based Approaches

### 8.1. Multi-Target Approaches

#### 8.1.1. Csy4 Nuclease Based Multi-Target Genome Editing

Multiplexed gene editing involves the multiple gRNA expression cassettes assembly, separate promoter involves to transcribe each (Figure 1). Typically, for expression of each gRNA Pol III promoters are used but the major limitation of Pol III is that it requires specific nucleotide at the 5’ end of the transcript. Due to multiple promoter sequences, the final array size can be unmanageable. To overcome this limitation multiple gRNAs can be expressed from a single transcript using Pol II promoter. The RNA-cleaving enzymes processed polycistronic mRNAs post-transcriptionally into individual gRNAs. These RNA-cleaving enzymes consist of endoribonuclease Csy4 (also called Cas6f) from *Pseudomonas aeruginosa* which belongs to subtype I-F CRISPR. With specific structure and sequence Csy4 (21.4 kDa) protein recognizes its RNA substrate, which cleaves pre-crRNA at 3’ end of the five base pair stable stem loop encoded by the CRISPR repeat, generating crRNAs which comprises of a distinctive spacer sequence which is flanked by 20 and 8 repeat derived nucleotides at 3’ and 5’ ends respectively [105]. Cermak et al. [106] performed the experiment to access the mutagenesis frequency of csy4 and tRNA processing enzymes using serval vectors. The first vector in which individual pol III promoters was used for each gRNA expression, single transcript with gRNA was produced by remaining vectors, separated by 71 bp tRNA^Gly^gene, 20 bp Csy4 hairpin or 15 bp ribozyme cleavage site. To direct the polycistronic mRNAs expression Pol II promoter was selected. Interestingly the result showed approximately two-fold higher frequency of mutagenesis with Csy4 and tRNA when compared to construct with pol III promoter. Number of CRISPR/Cas9 multiplex genome-editing tools was reported in cereal crops, however, these tools showed varied efficiencies, and generated multiple targeted mutations, suggesting CRISPR/Cas9-mediated quantitative traits improvement of crop by implementing multiplex genome editing system.

#### 8.1.2. Polycistronic t-RNA Transcripts Based Multi Target Genome Editing

The endogenous mechanism of tRNA processing in eukaryotes was used efficiently to achieve multi target genome editing. The tRNA processing mechanism cleaves tRNA precursor at both ends by recognizing specific sequence signatures. The present robust platform is implemented to increase the targeting and multiplexed genome editing without additional RNase together with Cas9/gRNA cassette. In plants, CRISPR/Cas9 system editing efficiency was improved by using this strategy. For simultaneous production of various gRNA, to target the different genes Xie et al. [20] developed a polycistronic tRNAs-gRNA (PTG) cassette. This PTG cassette transcribed as a common sgRNA gene under the regulation of Pol III promoter and consists of tandem repeats of tRNA-gRNA. Later the RNase P and RNase Z (in plants) of the endogenous nature tRNA processing RNase would recognize the tRNA components from the PTG transcript and excise individual gRNAs. This individual gRNAs with 5’ targeting sequence direct Cas9 for genome-editing at multiple target sites. In maize Qi et al. [107] optimized and introduced this strategy. Glycine-tRNA of Maize was used for multiple tRNA-gRNA units design under the control of maize U6 promoter for the simultaneous production of numerous gRNAs. This experiment showed single gene targeting with two different gRNAs using tRNA base approach which significantly enhances the efficiency of mutation in maize.

#### 8.1.3. Drosha MiRNA Based Multi Target Genome Editing

MicroRNAs (miRNAs) constitute a novel, phylogenetically extensive family of small RNA. These miRNAs are small single stranded RNAs of 22–25 nucleotides in length and have been discovered in plants and animals. Maturation of miRNA is a stepwise process catalyzed by RNase III type endonucleases such as Drosha and Dicer which contain dsRNA binding domain and catalytic RNase III domain. Drosha is required for processing of miRNAs precursors. miRNA transcripts or pre-miRNAs are first processed by Drosha in the nucleus then this precursor is exported to cytoplasm and further processed by Dicer. This mechanism of Drosha is used for multiplexed genome editing. To silence the target gene expression mediated through RNA interference (RNAi) an artificial RNA molecule having tight hairpin i.e., short hairpin RNA (shRNA) was used. Depending upon the promoter used in the assembly ShRNA can be transcribed by polymerase II or polymerase III. The transcribed product is processed by Drosha and it mimics pre-microRNA. By using the processing strategy of shRNA Yan et al. [108] developed sgRNA-shRNA structure which contains array of sgRNA-shRNA with interval sequence of Drosha cleavage site under the control of U6 promoter. After transcription, the transcript was processed by endogenous Drosha into individual sgRNA and shRNA. Later Cas9 directs the individual sgRNA to their respective target sites.

## 9. In the Absence of Integration Gene Expression and DNA Transfer

### 9.1. T-DNA Approach

Plant cells exchange DNA more often causing integration of transgene into the genome of host. On the other hand, the presentation of genes devoid of consequent integration for HDR is essential, transient expression of genome editing tools and genes critical for developmental reconstructing amid regeneration. To wipe out integration more such easly we all have to see how in plant genomes *Agrobacterium* coordinates with T-DNA. For the T-DNA joining *Agrobacterium* and plant genes both are vital, however lesser information regarding to prevent the integration how to control these genes are available. A mutant VirD2 protein harboring *Agrobacterium* strain is somewhat inadequate in delivery of T-DNA transiently, however seriously insufficient in the integration of T-DNA. Consequently, synthetic VirD2 which is non-integrating showed ideal transient expression characteristics may be generated for effective T-DNA delivery without integration. For the particle bombardment purpose, well-designed gold nanocomposites or a chemically coated particle which checks the release of DNA inside the nucleus of cell possibly will make possible transient nuclear expression in absence of integration of a transgene. Single stranded DNA barrage has been utilized as a procedure to maintain a strategic distance from template integration amid HDR-intervened genome altering [9]. Nonetheless, the template design and delivery determinations require advancement for reproducibility crosswise over various species. For the counter selection against events integrated transient expression system can be supported by selectable markers and reporter genes adjustment. DNA templates and devices for genome altering should be intended for self-extraction of events integrated arbitrarily. Genome editing of DNA-free nature is a predictable methodology for genomics research about and propelled plant breeding [109]. Plant breeders frequently wants specific mutations in a particular sequence of DNA without going with transgenic impression in the genome. Hence, without DNA genome altering approaches are appealing on numerous dimensions.

### 9.2. Cas9 Alternatives for More Precision

Currently, variants of Cas9 are used for more precise genome editing. Strategies such as Cas9 nickase (nCas9), dead Cas9 (dCas9) and chimeric Cas9 with Fok1 cleavage domain are used to reduce off target effects and enhance HR. Cas9 enzyme contains RuvC and HNH conserved nuclease domain which cleaves DNA strand non-complementary and complementary to the gRNA respectively. Cas9 nickase is produced by mutating catalytic residues (D10A in RuvC and H840A in HNH) which cleave only single strand of target DNA resulting nick in the single strand [8]. Use of this nCas9 with two different sgRNAs having close target site which will makes close nick in opposite strands results in DSB that can be repaired by NHEJ which leads mutation in target site thereby minimizes off target activity. Fusion of dCas9 with FokI monomer (fCas9) creates an RNA guided nuclease which cuts DNA only when two gRNAs binds to nearby regions with appropriate spacing and orientation, thus reduce off target cleavage. Guilinger et al. [110] observed 140-fold lower off-target/on-target modification ratio compared to wild type Cas9 additionally 1.3 to 8.8-fold lower than Cas9 nickase.

## 10. Preferred Promoters and Methods of Transformation for Genome-Editing of Cereals

### 10.1. Preferred Promoters for Gene Expression Regulation in Cereals

As discussed in above sections, for the controlled expression of CRISPR modules numerous kinds of promoters are being utilized. Such promoters are broadly categorized as constitutive promoters, tissue or developmental stage specific promoters, inducible promoters and synthetic promoters.

### 10.2. Constitutive Promoters

Constitutive promoters express downstream gene in all tissues continuously during different developmental stages. The constitutive promoters normally works across the different species and expression of these constitutive promoters are generally not adapted for endogenous elements. Typical examples of constitutive promoters include Cauliflower mosaic virus (CaMV) 35S, plant ubiquitin (Ubi), opine promoters, rice actin 1 (Actin-1) and maize alcohol dehydrogenase 1 (Adh-1) [111] Of these promoters, maize ubiquitin (Ubi) [112] and CaMV 35S [74] are mostly preferred for the expression of Cas protein. However, better and novel alternatives are still desirable. Park et al. [113], have analyzed *APX*, *SCP1*, *PGD1*, *R1G1B*, and *EIF5* for effective expression in rice. These promoters can provide efficacious alternatives to the generally used promoters in cases where they are not effective.

### 10.3. Promoters Specific for Tissue or Developmental Stage

Promoter regulates tissue(s) specific gene expression or at specific development phase have wide utility in genetic engineering. Thorough functional analysis of genes via regular gene knockout method is impeded because of the pleiotropic effects. Therefore, tissue or organ specific or developmental stage specific gene knockouts are more desirable. For instance, germline specific expression of Cas9 or other nucleases will reduce other adverse effect of these proteins during the plant development. Germline specific promoters, such as EC1.2 [114], SPOROTCYTELESS [114], *AtDMC1* [115], *Lat52* [116], and *DD45* [114], can especially be helpful to ensure the generation of heritable mutations. In this context, Decaestecker et al. [117] have developed a toolkit named CRISPR-based tissue-specific knockout system (CRISPR-TSKO) for Arabidopsis. The similar approach can be efficiently used in the genome editing of cereal crops.

### 10.4. Inducible Promoter

Inducible systems are generally composed of a chimeric transcription factor (the activator) under the control of a ubiquitous promoter and having the capacity to bind specifically to the target promoter. The functioning of inducible promoters depends upon environmental factors and exterior stimulators which can be artificially managed. Various abiotic stress factors such as heat, cold, oxygen level, light and wounding regulates the inducible promoters. Some components are hard to regulate through exploratory adjustments, the chemical compounds responding promoter, not found normally in the organisms are specifically compelling. Additionally, promoters that react to antibiotics, steroids, alcohol, copper, and herbicides have been adjusted and arranged to permit the initiation of gene action freely and autonomously against different abiotic or biotic stress factors. Glucocorticoid receptor (GR) based, GVG, AlcR/AlcA (ethanol inducible), and pOp/LhGR (dexamethasone inducible), and XVE/OlexA (beta-estradiol inducible) are some of the examples of inducible systems for plants [116]. However, these have not been used in for CRISPR/Cas till now, but offer a vast potential in cases where constitutive gene expression is not required.

### 10.5. Synthetic Promoters

Synthetic promoters have been developed by the amalgamation of various primary elements having diverse origins. On the basis of transactivating proteins synthetic promoters are regulatory expression systems. These promoters regulate gene expression irrespective of the physical position of the gene of interest. Constitutive promoters are regulatory system part in several promoters induced by chemicals, and incorporate transactivating proteins. Transactivating proteins comprise a completely separate area of molecules in the field of gene regulation which requires a separate study.

Most of the CRISPR/Cas9 constructs utilizes U6 or U3 which is a RNA Polymerase III promoter for driving sgRNA expression in monocots. Some of the features which make these promoters widely applicable for the expression of sgRNA, include the absence of downstream transcriptional initiation sites and distinct transcription initiation sites, close to universal expression. Examples of such synthetic promoters used in cereals have been cited in Table 2. Recently, in rice, U6 or U3 driven sgRNAs editing efficiency mediated through CRISPR/Cas9 was compared [115,118], which demonstrates that 21.5–45.6% enhancement in editing efficiency by U6 promoter compared to the U3 promoter were obtained. In case of multi target genome editing, Pol III promoters were wildly used for the expression of sgRNAs in a polycistronic tRNA-sgRNA [119]. Processing of these polycistrons within cells involves cleavage of tRNA stem-loop by RNases, leaving functional sgRNAs. U6 promoter of *Arabidopsis* and *O. sativa* was used successfully in *N. tabacum* and *S. bicolor* respectively [120]. Even though large number of naturally occurring as well as synthetic promoters are available, more number of such novel promoters will be needed to broaden the use CRISPR/Cas based genome editing. A positive correlation involving expression level of Cas9 and mutation frequency in rice calli was identified recently, which suggest that higher rates of gene editing events are the result of active promoters used. This possibly will be specifically important in stable transformation purpose where in the genome has an extended exposure time with sgRNAs and Cas9 proteins. Usage of bidirectional promoters is another possibility in this emerging genome editing field [121].

## 11. Transformation Methods for Genome Editing

**Figure 2:**
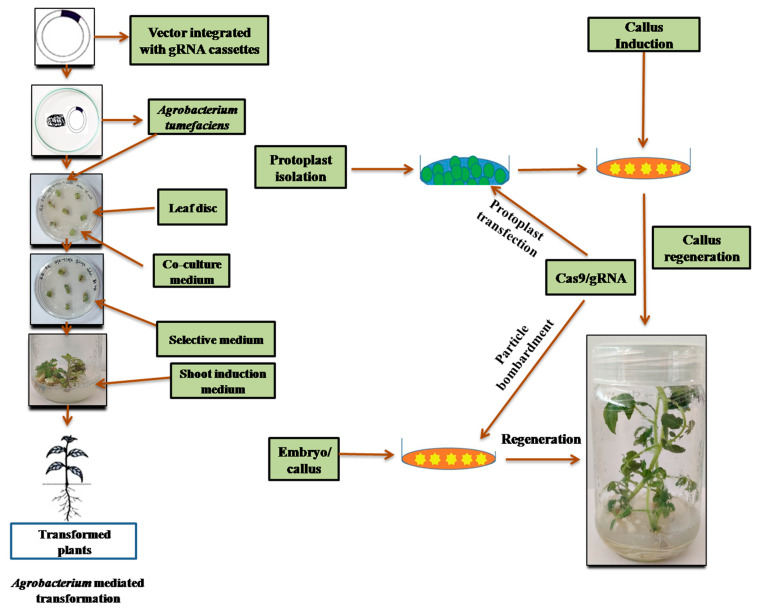
A simplified flowchart representation of steps involved in CRISPR/Cas9 mediated genome editing. The very first step involves the construction of vector using gRNA cassette followed by protoplast or *Agrobacterium* mediated plant transformation using various methods. After delivery into plants screening of putative transformants can be done using various methods..

Three main transformation methods are available presently, these includes *Agrobacterium*-mediated, particle bombardment, and protoplast transformation, methods. Out of the three mentioned methodologies one i.e., the method mediated through *Agrobacterium* is considered to be the most easiest and convenient. However, large number of horticultural crops cannot be transformed through *Agrobacterium* transformation method as they are not susceptible to the *Agrobacterium*. Through the particle bombardment and protoplast transformation methods host-dependent specificity of the *Agrobacterium*-mediated method can be overcome. However, shortcoming is also present in these methods, for example the particle bombardment method needs particular facilities and the handling skills are most important for the protoplast method. However, followed by plant transformation whole plant regeneration from a single cell is an alternative approach for many important horticulture crops (Figure 2).

### 11.1. Methods to Validate the Construct

#### 11.1.1. Protoplast

Fast and affordable method for the screening of genome edited plants is very important to save time and cost involved in tissue culture and regeneration of transgenic plants. To confirm genome edited efficiency, transfection of plant protoplasts provides fast option. Transfection of protoplast with genome editing reagents expressing plasmids mimic the in vivo conditions and provide idea about the efficiency of genome editing. Apart from this, protoplast transfection is most suitable to use as a high-throughput method. However, the method is not yet well optimized for many crop species.

#### 11.1.2. Protoplast Transfection

Protoplast are being used as a transient assay to test the genome editing efficiency as well as to identify on-target and off-target mutation frequency. Mostly enzymatically processed protoplasts of the leaf mesophyll are extracted and constructs of DNA or different biomaterials are delivered either through electroporation or PEG-interceded transfection. Similarly, different constructs of DNA having either circular or linearized plasmids, or expression cassettes of DNA, can be delivered together in the protoplast. Effective use of protoplast transfection has been previously demonstrated for genome editing using different methods such as ZFNs, TALENs and CRISPR/Cas9. The protoplast transfection is achieved in several cereals and model monocot plants including *Brachypodium*, wheat, maize and rice [48]. Transient assays are very important for the molecular biology experiments which facilitate functional annotation of genes, estimation of genome editing efficiency as well as understanding of the technological precision. For instance, in tobacco protoplasts ZFNs and a donor DNA template were transferred by electroporation, and 10% homologous recombination for altering the gene selected was achieved. Shan et al. [13] transferred TALENs into rice protoplasts targeting four genes by PEG-intervened transfection and mutations were generated as expected. Zhou et al. [89] additionally showed deletion of a large DNA segment in rice chromosome 2 targeted by a combination of various sgRNAs between two loci, and deletion of a cluster of 10 labdane-related diterpenoid synthetic genes (around 245 kb). Genome editing of plants based on protoplast was analyzed utilizing most altering techniques which include both interferences of gene (deletion and addition) and substitution of gene through the mechanisms of NHEJ or HDR.

#### 11.1.3. Agroinfiltration Methods

A transient expression assay mediated through *A. tumefaciens* mostly applied for dicot plant is called as *Agroinfiltration*. In this assay, plant leaves are used to infiltrate the *Agrobacterium* as a liquid culture, which results in transgenes transfer into the cells of plant from the bacterial Ti plasmid T-DNA region. Transgene is expressed in the infiltrated region of most of the plant cells. A DNA fragments (>2 kb) length can be used and can deliver multiple transgenes in the same cell. Co-expressing transgenes might be available in numerous *Agrobacterium* cultures, necessary steps prior to infiltration is mixing, multiple binary vectors or multiple genes in single binary vector can be carried by single *Agrobacterium*, this methodology was largely exploited to check in vivo mutagenesis [122]. By applying agroinfiltration in *N. benthamiana* leaves Mahfouz et al. [123], reported that a DSB develops by Hax3 TALE-based hybrid nuclease in target sequence. *pcoCAS9* and *AtPDS3* were co-expressed by Li et al. [5] in a single binary plasmid in leaves of *Arabidopsis* and *N. benthamiana*, and demonstrated mutations in both species in the two target sequences.

#### 11.1.4. Hairy Roots Validation

In plants, hairy roots induced by *A. rhizogenes* has been used to evaluate genome editing efficiency in many plant species. For instance, Curtin et al. [124] employed hairy root transformation techniques to evaluate efficiency of genome editing using ZFN approach performed for nine endogenous soybean genes namely *DCL1a*/*DCL1b*, *DCL4a*/*DCL4b*, *DCL2a*, *DCL2b*, *RDR6a*, *RDR6b*, and *HEN*. Comparative efficiency of TALEN and CRISPR/Cas technologies were studied by Du et al. [125] by targeting two genes *GmPDS11* and *GmPDS18* in soybean hairy root system.

#### 11.1.5. High Precision Base Editing

Precision base editing is a genome modification strategy that legitimately creates specific point mutation without DSBs generations in the DNA (genomic) or in cell RNA. It requires donor DNA template depending on cell HDR. Precision base editing include combinations between Cas nuclease and enzymes modifying bases, which function on single stranded DNA. When binds to target locus in DNA, shifting of single stranded DNA segment in a ‘R loop’ takes place due to the pairing of bases between gRNA and the targeted DNA strand. To enhance the effectiveness in eukaryotic cells, a nick also generated by the catalytically disabled nuclease, which induces cells strand repair without the editing of bases by means of utilizing the strand edited as a template. Editing of cytosine base change a C•G base pair into a T•A base pair, and editing of adenine changes A•T base pair into a G•C base pair, these are the two classes of base editing which has been explored widely [126].

### 11.2. Gene Replacement

Along with the Cas9 nuclease TALENs or sgRNAs pairs were used to achieve the targeted genomic deletion. For targeted gene replacement, a prerequisite step is generation of double strand break lessions or mutations generated due to deletion. In protoplasts of tobacco (*N. benthamiana*) HDR-mediated gene replacement was successfully achieved through CRISPR/Cas9 in in vitro system [42]. In recent times replacement of DNA/gene with accuracy becomes an effective tool for genome-editing, those are greatly required for breeding and molecular engineering purposes. Despite the fact that the CRISPR/Cas9 system functions in plants as a gene knockout device, the replacement of gene has seldom been accounted. The *MIR169a* and *MIR827a* (miRNA gene regions) were deleted successfully by Zhao et al. [127] by using combinatory dual-sgRNA/Cas9 vector designed first time and further confirmed by PCR followed by sequencing, resulted with 20% and 24% efficiencies of deletion on *MIR169a* and *MIR827a* loci were obtained respectively.

### 11.3. Gene Expression Modulation

Regulation of gene expression is much important for genetic engineering, synthetic biology and applied studies. Many genetic tools are available for endogenous gene expression silencing or knocking down or enhancement of expression. In the early 2000s, artificial transcription factors which is based on zinc finger, and transcription activator-such as effector transcription factors have been used to modulate gene expression [128]. The emergence of more advanced and broadly accepted CRISPR/Cas9 systems for transcriptional regulation open the path for controlling the expression of gene endogenously by utilising various transcription factors. The most important advancement of CRISPR/Cas9 is the capability to regulate control of endogenous transcription. The critical impact of Cas9 proteins, that is linked to transcriptional effector domains is able to activate or repressed the genes. Simplistic and accountable multi gene targeting ability is the characteristics of CRISPR systems, simultaneously expressing multiple gRNAs in a given cell, gives the capacity to accomplish multiple transcriptional activations [129].

## 12. Challenges for Genome-Editing in Cereals

### 12.1. Polyploidy

Acquiring one or additional complete chromosome sets within an organism is categorized as Polyploidy. At least three classes of polyploidy exist viz. autopolyploids, allopolyploids, and segmental allopolyploids. Polyploidy genomes are complex in many ways. They are difficult to sequence due to repetitive sequences as well as larger genome size. The higher number of gene copies, and different functions of duplicated genes evolved through neofunctionalization also make it difficult for functional annotations. Such complexity makes it challenging to achieve desired mutations. Sometimes mutation particularly knockdown or knockout of gene may result in no phenotypic change due to dose effect of other paralogous copies of genes. Such issues are also faced while perusing a genome editing in polyploidy crop such as wheat. Apart from such obvious issues, multiple copies of genes make it difficult to achieve editing in any specific copy. On the other hand sometimes desired trait manipulation needs editing of all the paralogs which can hamper the efficiency greatly [130,131]. In a recent study performed in tetraploid oilseed rape Braatz et al. have stably transformed two *ALCATRAZ* (*ALC*) homoeologs using CRISPR-Cas9 construct. Additionally using single target sequence they obtained transgenic T1 plant with four *alc* mutant alleles. The concurrent editing of multiple homoeologs through CRISPR-Cas9 mutagenesis without any background mutations will provide new possibility to use mutant genotypes in breeding [132].

### 12.2. Transformation Efficiency

Genome-modification for improvement of important crop is an uncommon innovative revolution, however, still there are major limitations to its execution. Characterizing genetic variations and genetic regulation studied in different crop species and model plants have broadened our understanding which can be efficiently explored to modify desired genes in crop system. Exploration of recent advancement in plant genome editing techniques for the improvement of any specific crop species largely depends on transformation efficiency. Two distinct and consecutive steps of plant transformation are: (1) transient transformation (introduction of DNA in plant cells which is not heritable), and (2) stable transformation (integration of DNA into the plant genome which can be stably inherited to next generation). Both progressions are valuable for plant biotechnology and fundamental research however, the second one is important to develop transgenic plants having heritable genetic modifications. For the stable transformation, the step of regeneration is more prominent limitation compared to the stable integration of the transgene. Over the last three decades, transformation mediated through *Agrobacterium* and particle bombardment have been widely used in plant science. Although it is used widely still not efficient for many crop plants. The difficulties arises are (1) for generation of plants carrying transgene through tissue culture is highly time consuming, (2) events transformed stably showed lower frequency, (3) titre of DNA is lower, and (4) gene transfer mediated through particle bombardment exhibits less precision. For crop plant improvements simplification of the transformation protocol being used currently would be the model resolution that could be utilized in many labs. Advancements in each area should be in such a way that it increases intact single-copy expression cassettes delivery with reduced damage of plant tissues. Extended study should be carried to magnify the plants transformation and regeneration responses by targeting a wide range tissues and genotypes [133].

## 13. Off-Target Effect with Cas9 and Improved Variant of Cas9

The major issue related to CRISPR/Cas9 mediated genome editing is the off-target effect of Cas9. It can cause unwanted cleavage in the genome. A higher number of off-target events can induce toxicity at the cellular level which is not a favorable condition for plants. Also, the undesired chromosomal rearrangements such as deletions, inversions, and translocations, caused by the repair of these off-target DSBs can be harmful to plants [133,134,135]. However, in plants, mild off-target activity is reported as compared to animals. The specificity of Cas9 is delimited by 20 nucleotide guide sequence of sgRNA and PAM sequence. Many reports show the presence of off-target DNA cleavage with one to five bp mismatches in sgRNA sequences. It is reported that PAM sequence plays a role in the binding of Cas9 while 3’ end is critical for target identification R-loop formation, and activation of nuclease activities in Cas9, some mismatches at 5’ end are bearable [136]. The two major factors controlling the on-target and off-target cleavages are the composition of guide RNA, as well as the structure of guide RNA. By manipulating the composition and structure of sgRNA one can reduce the off-target events. Using different approaches this can be achieved, significantly low or high GC content makes sgRNA less eventful [137], truncation of sgRNA at 5’end or 3’end, by using sgRNA nickase [138]. Depending on the GC content of seed sequence the efficient sgRNA can be selected to carry out CRISPR/Cas9 mediated genome editing with minimal off-target events [139]. Various methods have been reported to examine off-target events including *in silico* prediction, T7E1 assay, HTGTS, ChIP-seq, IDLV, fluorescence in situ hybridization, deep sequencing, etc. Among these techniques, Digenome-seq and GUIDE-seq emerged as a precise method for off-target identification with 0.1% sensitivity.

The studies suggest that the incident of stable mutation transmission in successive generation using CRISPR/Cas9 is higher in rice than Arabidopsis [140]. When Arabidopsis plants mutated using CRISPR/Cas9 it was observed that somatic mutation in T1 generation did not pass through T2 generation while germline mutation present in T1 generation was passed to T2 and T3 generation as such as Mendelian inheritance [141]. These results suggest that mutation occurred in germline is stably integrated into the later generations. Mao et al. [114] used Pollen-specific promoter to develop CRISPR/Cas9 construct which resulted in development of heritable biallelic T1 mutants. So, it can be predicted that using germline specific promoters one can achieve expected Cas9 expression with increased germline mutation which can result in stable transmission in the future generation.

To reduce the off-target effect different Cas9 variants have been developed. Guilinger et al. [110] merged dCas9 with FokI nuclease to develop fCas9, fCas9 derived modified cells showed 140 fold increased specificity. Kleinstiver et al. [55] carried out 3–4 amino acid substitutions in Cas9 which resulted in no detectable off-targets. These Cas9 variants can be replaced with Cas9 to overcome the issue of off-target events.

## 14. Regulatory and Ethical Issues

Recent and rising gene editing methods make it conceivable to focus genes of interest in particular species with more noteworthy speed and effectiveness compared to the conventional methods [142]. Of significant pertinence for plant breeding, controllers and researchers are talking about how to direct developed products utilizing these editing techniques of gene. Such discourses incorporate whether to carry or adjust the present system for GMO risk regulations in assessing the effects of gene edited plants, and related products, living organisms, environment and society. Classification of generated products is one of a few parts of the present structure being condemned. Further, information gaps identified with risk evaluations of gene edited living beings, for instance of target and off-target impacts of intervention in plant genomes are additionally of concern. Settling these and related issues of the present system will include tending to numerous subjective, esteem loaded situations, for instance how to indicate insurance objectives through environment administration approaches. A procedure educated by capable research and advancement works, including a more extensive network of individuals, associations, specialists, and intrigue gatherings, could support researchers, regulators, and different partners address these complex, value-laden concerns identified with gene editing of plants for society [143]. Similar to other genome editing, possibility of inducing mutations, in the genome off-target mutations may be induces by CRISPR/Cas9 system [91]. During different stages of embryonic development CRISPR/Cas9 system repetitively target genes, which results in Mosaicism of the mutation(s) [92]. An unintended mutation take place once DNA sequences within the genome are cleaved by CRISPR-Cas9, those are homologous to the target DNA sequences, causes, off-target mutations which could be lethal, may cause the death of the cell or transformation [57]. An additional concern is to expand the effectiveness of homologous recombination and homozygous knockout. The most important dispute is to make sure the effectiveness of genome editing without introducing alteration in undesired parts of the genome. Present methods are advanced enough to target precisely the desired locus without any off-target effects. However, such technological advances have not been explored sufficiently.

Concerning the difficulties related with risk regulations of emerging gene editing methodology, there are no specific guidelines. Present guidelines being followed for commercial release of GMO are widely considered as a base for the genome edited plants. European administrative necessities that release of GMOs, related foods and feeds are set up in EU Directive 2001/18/EC (initially 90/220/EC), in the guideline (EC) No. 1829/2003 and its sister guidelines, just as in different national systems. Key to any administrative prerequisite is a component of evaluating risks to human, animal, and ecological wellbeing. At pan-European-level, such hazard evaluations depend on a case-by-case process and through an organised strategy. The European Food Safety Authority (EFSA) gives scientific surveys and appraisal of security and GMOs effect on environments, however, the European Commission is answerable for decisions related with risk management of GMOs [144]. National concerns to the developing utilization of new and rising techniques based on gene editing in plants bring up issues of whether such advancements (a) may be absolved from current GMO guidelines, as well as (b) if existing guidelines require correction and adjustment to properly oversee new products coming from these techniques [145]. As mentioned, the principle contention for exclusion from current GMO guideline is the closeness of organisms modified with new and rising gene editing method to organisms coming from chance mutagenesis. The justification of exclusion depends on relatedness conceive that organisms developed through gene editing are impossible to differentiate from generated products through previously exempted methods [146]. A significant supposition of this contention is that any hazards linked with this rising techniques of gene editing will be moreover close and equivalent to, or less noteworthy comparatively menace linked with excluded procedures or products [147].

## 15. Conclusions

In last decades CRISPR become one of the most versatile genetic engineering tool that has been utilized for various application of genome editing. Genome editing approaches are more cost efficient, quicker, and precise in achieving desired crop improvement as compared to traditional methods and transgenic approaches. Still genome editing faces many challenges in their application, and to enhance their applicability in cereals and other crops there is need of removing these barriers to promote the effective implementation of these genome editing techniques for crop improvement with future prospects. Out of the many challenges CRISPR-based genome editing technique faces, the major one is the transformation efficiency which needs to be optimized for different cultivars. In case of most of the cereal crops, transformation protocol is already developed but such protocols are largely genotype specific. In such scenario, genome editing cannot be explored efficiently in the genetic background of high yielding commercial cultivars. Other minor concerns such as the requirement of PAM (for many Cas variants) that may make it difficult to perform genome editing for a gene lacking the particular PAM sequence. In spite of availability of these important genome editing techniques, still there is certain restriction in the application of CRISPR tools for the agricultural crops improvement due to certain global regulation. By overcoming the restriction of CRISPR techniques for the improvement of crops of agricultural importance, the productivity of new and healthier food can be enhanced. Crop plants generated through CRISPR/Cas9 genome editing might be established as non-genetically modified organism, for its fast acceptance at field level. We can anticipate that the implementation of CRISPR/Cas9 technology in agriculture will revolutionize the crop productivity and tend to be a second green revolution that will ensure ever rising populations of world for their food and nutritional requirement.

## Figures and Tables

**Table 1:** List of significant gene editing studies in cereal crops.

Plant Species	Delivery Mode	Target Gene(s)	Gene Function	Vector Used	Promoter Used	Reference
Maize	*Agrobacterium-*transformation	*ALS2*	A key enzyme for the biosynthesis of branched-chain amino acids (major targets for herbicides)	UBI:Cas9 T-DNA vector	ZmU1	[9]
Maize	*Agrobacterium*-mediated transformation	*PSY1*	Phytoene synthase	pMD18-T	ZmU6	[10]
Maize	Protoplast transformation	*Zmzb7*	Encodes IspH protein for methyl-D-erythritol-4- phosphate (MEP) Pathway	pEasy-Blunt simple vector	ZmU3	[11]
Maize	*Agrobacterium*-mediated transformation	*ARGOS8*	Increased grain yield under drought stress	sgRNA-Cas9	ZmU6	[12]
Wheat	*Agrobacterium*-mediated transformation	*TaMLO*	Mildew resistance locus	pUC-T vector (CWBIO)	TaU6	[13]
Wheat	Biolistic transformation	*TaMLO-A1*	Powdery mildew resistance negative regulator	pJIT163	TaU6	[14]
Wheat	*Agrobacterium*-mediated transformation	*TaVIT2*	Fe content	p416-MET25	HMW-GLU	[15]
Wheat	Biolistic transformation	*TaEDR1*	Disease resistance Against powdery mildew	pJIT163-Ubi-Cas9	TaU6	[16]
Wheat	Biolistic bombardment	*TaGW2*	Grain weight negative Regulator	pET28a-Cas9-His	TaU6	[17]
Wheat	Biolistic transformation	*Alpha-gliadin*	Low-gluten	pANIC-6E	TaU6	[18]
Rice	*Agrobacterium*-mediated transformation	*osPPKL1*	increases length and yield	pCAMBIA1300S	CaMV 35S	[19]
Rice	*Agrobacterium*-mediated transformation	*OsMPK5*	Various abiotic stress tolerance and disease resistance	pRGE3 and pRGE6	OsU3 and OsU6	[20]
Rice	*Agrobacterium*-mediated transformation	*TMS5*	Negative regulator of thermo-sensitive genic male sterility	TMS5as	OsU3	[21]
Rice	*Agrobacterium*-mediated transformation	*ALS*	A key enzyme for the biosynthesis of branched-chain amino acids, (major targets for herbicides)	pCXUN-Cas9-gRNA1-gRNA2-armed donor vector	OsU3	[22]
Rice	Biolistic transformation	*EPSPS*	A key enzyme of aromatic amino acids biosynthesis	pHUN411	OsU3	[23]
Rice	*Agrobacterium*-mediated transformation	*CSA*	Negative regulator of photoperiod-sensitive genic male sterility	CH-CRISPR/Cas9-CSA, Gateway-CRISPR/Cas9-CSA	OsU6a	[24]
Rice	*Agrobacterium*-mediated transformation	*DEP1*	Regulators of inflorescence Architecture of plant height	pYLCRISPR/Cas9(I)	OsU6a	[25]
Rice	Electroporation	*ERF922*	Rice blast resistance negative regulator	C-ERF922	OsU6a	[26]
Rice	*Agrobacterium*-mediated transformation	*NRT1.1B*	Nitrogen transporter	PCSGAPO1	OsU6	[27]
Rice	*Agrobacterium*-mediated transformation	*OsHAK-1*	Low cesium accumulation	pH-Ubi-Cas9-7	OsUbi	[28]
Rice	Electroporation	*SBEIIb*	high-amylose	pCXUNCas9	OsU3	[29]
Rice	*Agrobacterium*-mediated transformation	*OsNramp5*	Low Cd-accumulation	pYLCRISPR/Cas9Pubi-H	OsU3, OsU6	[30]
Rice	*Agrobacterium*-mediated transformation	*eIF4G*	Resistance to rice tungrospherical virus	pCas9-eIF4G-gRNA	TaU6	[31]
Rice	*Agrobacterium*-mediated transformation	*OsPRX2*	Potassium deficiency tolerance	pCAMBIA1301	OsPRX2	[32]
Rice	*Agrobacterium*-mediated transformation	*Waxy*	Amylose content	CRISPR/Cas9 vector	OsU6	[33]
Rice	*Agrobacterium-*mediated transformation	*NGv1*	Reduction of off-target effects	APOBEC-UGI	OsU6	[34]
Rice	*Agrobacterium-*mediated transformation	*Tos17*	retrotransposon	CRISPR/Cas9 vectors	OsU6	[35]
Rice	*Agrobacterium*-mediated transformation	*ISA1*	isoamylase-type debranching enzyme	VK005	OsU6	[36]
Rice	*Agrobacterium*-mediated transformation	*OsRR22*	salinity tolerance	pYLCRISPR/Cas9Pubi-H	OsU6	[37]
Rice	*Agrobacterium*-mediated transformation	*OsITPK6*	Low phytic acid	pHun4c12s	OsU6	[38]
Barley	*Agrobacterium*-mediated transformation	*HvPM19*	ABA-inducible plasma membrane protein	pAGM4723	TaU6	[39]

**Abbreviations:** CRISPR: Clustered regularly interspersed short palindromic repeats; Cas9: CRISPR associated protein 9; OsU: *Oryza sativa* small nucleolar RNA (snoRNA) promoters; sgRNA: single guide RNA; ZmU: *Zea mays* snoRNA promoters; TaU: *Triticum aestivum* snoRNA promoters.

**Table 2:** List of significant multi target genome-editing studies in cereal crops.

Plant Species	Delivery Mode	Target Gene(s)	Gene Function	Vector Used	Promoter Used	Reference
Maize	*Agrobacterium-* transformation	*ZmIPK1A*, *ZmIPK* and *ZmMRP4*	Phytic acid synthesis	pEasy Blunt vector	ZmU3	[62]
Maize	Biolistic-mediated transformation	*LIG, MS26, MS26, MS45, LIG, MS26, MS45*	LIG (liguleless) MS26 and 45 (male sterility)	Cas9 DNA vector	ZmU6	[9]
Maize	*Agrobacterium*-mediated transformation	*ZmLG1*, *UB2*, and *UB3*	Development of a haploid-inducer mediated genome editing system	pCPB	CaMV 35S	[63]
Wheat	PEG4000-mediated transformation	*TaDEP1, TaGASR7,* T*aLOX2, TaNAC2, TaPIN1, TaGW2*	Inflorescence architecture and plant height regulator, lipoxygenase, grain weight negative regulator	pJIT163	TaU6	[64]
Wheat	*Agrobacterium*-mediated transformation	*TaDREB2* and *TaERF3*	Drought resistance	pJIT163-2NLSCas9	TaU6	[57]
Rice	*Agrobacterium*-mediated transformation	*OsSWEET11, OsSWEET14*	sucrose efflux transporter	pTOPO/D	OsU6	[65]
Rice	Biolistic transformation	*OsBADH2, Os02g23823, OsMPK2*	Responsible for aroma, a basic helix–loop–helix (bHLH) transcription factor, a mitogen-activated protein kinase	pJIT163	OsU3	[13]
Rice	Biolistic transformation	*OsMPK2, OsDEP1*	Yield under stress	pJIT163	OsU3	[13]
Rice	*Agrobacterium*-mediated transformation	*OsDERF1, OsPMS3,* *OsEPSPS, OsMSH1, OsMYB5*	Drought tolerance	sgRNA-Cas9	OsU3	[58]
Rice	*Agrobacterium*-mediated transformation	*OsPDS, OsPMS3, OsEPSPS, OsDERF1, OsMSH1, OsMYB5, OsMYB1, OsROC5*, *OsSPP* and *OsYSA*	(*OsPDS*) pigment synthesis, (*OsEPSPS*) synthesis of aromatic amino acid, (*OsMSH1*) DNA mismatch repair protein, (OsROC5) Rice Outermost Cell-specific gene5, (*OsDERF1)* AP2 domain containing protein, (*OsYSA*) pentatricopeptide repeat domain containing protein	WDV2-ACT1 and WDV2-GST	35s	[25]
Rice	*Agrobacterium*-mediated transformation	*OsAOX1a,* *OsAOX1b, OsAOX1c, OsBEL*	Various abiotic stress tolerance	GATEWAY-based vector	OsU3	[66]
Rice	*Agrobacterium*-mediated transformation	*OsU3, OsU6a, OsU6b, OsU6c*	OsWaxy; amylase synthase	pCAMBIA1300	OsU3, OsU6b, and OsU6c	[67]
Rice	*Agrobacterium*-mediated transformation	*GS3, GW2, GW5, TGW6*	Grain Size 3 (*GS3*), grain width 2 (*GW2*), grain width 5 (*GW5*) and thousand-grain weight 6 (*TGW6*), negatively regulated grain weight	pHUN412	GW2-OsU3 GW5-OsU6 TGW6-TaU3	[6]
Rice	*Agrobacterium*-mediated transformation	*DEP1, Gn1a, IPA1, GS3*	(*DEP1*) inflorescence architecture and plant height, (*Gn1a*) grain number negative regulator, (*IPA1*) plant architecture regulator, (*GS3*) negative regulator of grain size	pYLCRISPR/Cas9(I)	OsU6a	[24]
Rice	*Agrobacterium*-mediated transformation	*GW2GW5* *TGW6*	Grain weight negative regulator	pHUN412 vector	OsU3, OsU6 and TaU3	[6]
Rice	*Agrobacterium*-mediated transformation	*PYL1–PYL6* and *PYL12(gp-1), PYL7–PYL11* and *PYL13(gp-2)*	best growth and improved grain productivity	PCAMBIA1300	OsU3, OsU6	[68]
Rice	*Agrobacterium*-mediated transformation	*SPO11-1*, *REC8*, *OSD1*, *MATL*	Introduction of apomixis	pC1300-Cas9	OsU6	[69]
Rice	*Agrobacterium-*mediated transformation	*BBM1, BBM2 and BBM3*	Redirection for asexual propagation through seeds	pCRISPR BBM	OsU6	[70]
Rice	Transformation by gene gun	*GUS, PDS, Chalk5*	Investigation of the efficiency of CRISPR/Cas9 in creating genomic deletions	pRGE32, pJU24, pJU34 and pJU46	OsU3	[71]
Rice	*Agrobacterium*-mediated transformation	*SWEET11*, *SWEET13* and *SWEET14*	resistance to bacterial blight	pBY02-ZmUbiP-OsCas9	ZmUbi	[72]
Rice	*Agrobacterium*-mediated transformation	*OsLCT1* and *OsNramp5*	Low cadmium (Cd)	pHun4c12s	OsU6	[73]

**Abbreviations:** CaMV 35S: Cauliflower Mosaic Virus promoter; Cas9: CRISPR associated protein 9; CRISPR: Clustered Regularly Interspersed Short Palindromic Repeats; OsU: *Oryza sativa* snoRNA promoters; sgRNA: single guide RNA; TaU: *Triticum aestivum* snoRNA promoters; ZmU: *Zea mays* snoRNA promoters; ZmUbi: *Zea mays* Ubiquitin promoter.
